# Occupational Licensing in Later Life: Work and Retirement Decisions Before and After the COVID-19 Pandemic

**DOI:** 10.1093/geroni/igaf122.2334

**Published:** 2025-12-31

**Authors:** Yun Taek Oh

**Affiliations:** University of Nevada, Reno, Reno, Nevada, United States

## Abstract

Attaining occupational licenses is considered a career development option for younger workers because the attainment cost outweighs the benefits for older workers. Contrary to this expectation, a significant proportion of workers attain licenses in their later lives. However, the reasons and outcomes of later-life attainment of occupational licenses are not well-documented. This study investigates and compares the sociodemographic and economic predictors of later-life attainment of occupational licenses and the post-attainment work and retirement decisions before and after the COVID-19 pandemic. Using the sample of respondents aged between 55 and 63 from the Current Population Studies 2018-2019 (men = 1,927 and women = 1,576) and 2023-2024 (men = 1,501 and women = 1,182), I first conduct logistic regressions to examine the predictors of later-life attainment. Then, I use clustered competing risk analysis with propensity score matching to compare the choice of retirement pathways of newly licensed and unlicensed workers in pre- and post-pandemic cohorts. The results show that men’s license attainment decreased (OR = 0.439) after the pandemic while women’s attainment increased (OR = 8.399). Furthermore, newly licensed women are more likely to move to different employers than unlicensed women before and after the pandemic (SHR = 1.50 and 1.23 respectively), while newly licensed men are more likely to switch to part-time than unlicensed men after the pandemic (SHR = 3.75). These results imply that the purpose of later-life attainment of occupational licenses varies by gender and period yet contribute to longer work lives by taking on alternative retirement pathways.

